# Diagnostic Accuracy of Machine Learning-Assisted MRI for Mild Cognitive Impairment in Parkinson's Disease: A Systematic Review and Meta-Analysis

**DOI:** 10.1155/padi/2079341

**Published:** 2025-05-22

**Authors:** Feng Zhang, Liangqing Guo, Lin Liu, Xiaochun Han

**Affiliations:** ^1^Department of Ultrasound, Affiliated Hospital of Shandong University of Traditional Chinese Medicine, Jinan 250014, Shandong, China; ^2^Department of Endocrinology, Affiliated Hospital of Shandong University of Traditional Chinese Medicine, Jinan 250014, Shandong, China; ^3^Special Examination Department, Affiliated Hospital of Shandong University of Traditional Chinese Medicine, Jinan 250014, Shandong, China; ^4^School of Health, Shandong University of Traditional Chinese Medicine, Jinan 250355, Shandong, China

**Keywords:** cognitive dysfunction, machine learning, magnetic resonance imaging, meta-analysis, Parkinson's disease

## Abstract

To evaluate the diagnostic accuracy of machine learning-assisted magnetic resonance imaging (MRI) in detecting cognitive impairment among Parkinson's disease (PD) patients through a systematic review and meta-analysis. We systematically searched for studies that applied machine learning algorithms to MRI data for diagnosing PD with mild cognitive impairment (PD-MCI). Data were extracted and synthesized to calculate pooled sensitivity, specificity, positive likelihood ratio (PLR) and negative diagnostic likelihood ratio (NLR), and diagnostic odds ratios (DOR). A bivariate random-effects model and summary receiver operating characteristic (SROC) curves were employed for statistical analysis. The quality of studies was assessed using the Quality Assessment of Diagnostic Accuracy Studies (QUADAS-2) instrument. The publication bias was investigated through Deeks' funnel plot. All statistical analyses were conducted using Stata 14.0. The pooled sensitivity and specificity for diagnosing PD-MCI using machine learning-assisted MRI were 0.82 (95% CI: 0.75–0.87) and 0.81 (95% CI: 0.73–0.87), respectively. The PLR was 4.28 (95% CI: 2.93–6.27), and the NLR was 0.23 (95% CI: 0.16–0.32), indicating a high diagnostic accuracy. The area under the curve (AUC) for the SROC was 0.85 (95% CI: 0.82–0.88). Quality assessment using the QUADAS-2 tool showed a predominantly low risk of bias among the studies, and the Deeks' funnel plot suggested no significant publication bias (*p*=0.30). In summary, the MRI combined with machine learning for diagnosing PD-MCI achieved high accuracy with the pooled sensitivity of 82% and specificity of 81%.

## 1. Introduction

Parkinson's disease (PD) remains a focal point due to its complex pathology and significant impact on patient quality of life [1]. Among its symptoms, cognitive decline, particularly mild cognitive impairment (PD-MCI), emerges as a critical concern [2]. PD-MCI, situated on the continuum between normal cognitive aging and dementia, poses a significant challenge in early detection and diagnosis, underscoring the urgent need for more accurate and efficient diagnostic methods [3]. This transition, documented in multicenter cohort studies, highlights the importance of identifying PD-MCI early to enable timely therapeutic interventions that can potentially mitigate cognitive deterioration [4]. However, the diagnosis of PD-MCI presents substantial challenges. Traditional diagnostic frameworks primarily depend on detailed clinical symptoms and neuropsychological assessments, which, while thoroughly, face limitations in homogeneity and practicality [5]. These assessments can be labor-intensive and may not always capture the nuanced changes specific to PD-MCI, leading to potential underdiagnosis. The condition's subtle impact on functional independence and daily life further complicates its identification, as patients and clinicians might overlook early cognitive symptoms [6]. Consequently, there is a pressing need for more straightforward, accurate diagnostic methodologies for PD-MCI.

Magnetic resonance imaging (MRI) plays a pivotal role in the diagnosis and understanding of PD-MCI, leveraging its capability to provide detailed insights into the brain's structure and function [7]. The mechanisms underlying PD-MCI characterized by the abnormal accumulation of Lewy bodies and β-amyloid (Aβ) in neurons, along with glial cell damage, demyelination, and increased microglial concentrations, provide a fundamental basis for utilizing MRI in its diagnosis [8, 9]. Diffusion tensor imaging (DTI), a form of MRI that measures the diffusion of water molecules in the brain tissue to assess white matter integrity, is particularly useful in this context [10]. In PD-MCI, demyelination of axons can disrupt the normal flow of water molecules, leading to changes in DTI metrics [11]. Quantitative susceptibility mapping (QSM), an MRI technique that quantitatively maps tissue magnetic susceptibility, has shown potential in detecting the microstructural changes associated with PD-MCI [4]. Studies have linked excess iron accumulation in deep gray matter nuclei—a feature quantifiable through QSM—to cognitive decline in PD [12, 13]. While these advancements offer significant insights, the exact cutoff values for distinguishing PD-MCI from normal cognition remain elusive, highlighting the need for more refined analytical approaches. Machine learning algorithms, known for their ability to identify patterns in complex datasets, offer the potential to refine the diagnostic process for PD-MCI [14]. By training models on MRI images, researchers aimed to develop a methodology that can accurately classify PD patients into PD-MCI and normal cognition groups [15]. This synergy between advanced imaging techniques and artificial intelligence promises not only to streamline the diagnostic process but also to provide deeper insights into the underlying mechanisms of cognitive decline in PD.

This systematic review and meta-analysis aimed to synthesize the existing evidence on the efficacy of MRI combined with machine learning in diagnosing PD-MCI. By evaluating the diagnostic accuracy of this approach, this work seeks to contribute to the development of more effective diagnostic strategies, ultimately facilitating early interventions and improving outcomes for patients with PD.

## 2. Materials and Methods

This systematic review and meta-analysis adhered strictly to the Preferred Reporting Items for Systematic Reviews and Meta-analyses (PRISMA) guidelines for diagnostic test accuracy studies [16]. The methodology encompassed a comprehensive literature search, study selection, data extraction, and statistical analysis, with the goal of synthesizing existing evidence on the subject matter.

### 2.1. Search Strategy

We systematically searched databases including PubMed, Embase, Cochrane Central Register of Controlled Trials (CENTRAL), and Web of Science, without language restrictions, from their inception to February 10, 2024. The search strategy was crafted to include a combination of keywords and MeSH terms related to “Parkinson's disease,” “cognitive impairment,” “cognitive dysfunction,” “MRI,” “artificial Intelligence,” “deep learning,” and “machine learning.” The detailed search strategy was presented in Supporting Table 1.

### 2.2. Inclusion and Exclusion Criteria

The inclusion criteria were as follows: (1) studies reporting machine learning-assisted MRI diagnostics for PD-MCI; (2) studies providing detailed information on patient demographics, study year, and diagnostic methods; and (3) studies offering data on diagnostic accuracy such as ROC curves, sensitivity, specificity, and diagnostic odds ratios. The exclusion criteria included (1) duplicate publications, (2) conference abstracts, (3) reviews, and (4) case report.

### 2.3. Study Selection

Duplicates were eliminated utilizing EndNote X9.0. Two researchers independently conducted searches for articles based on the previously described search strategy. Titles and abstracts of all located records were assessed for possible inclusion, adhering to the predefined inclusion and exclusion criteria. Subsequently, a comprehensive review of the full texts was undertaken for studies deemed potentially suitable. Discrepancies were addressed by discussion and reaching a consensus; if required, a third researcher participated in the resolution process.

### 2.4. Data Extraction and Quality Assessment

Two authors independently extracted data from the selected studies: including the author, publication year, country of origin, study design, participant gender, patient count, patient age, and the employed machine learning model. The Quality Assessment of Diagnostic Accuracy Studies (QUADAS-2) instrument, encompassing four domains (selection of participants, the index test, the reference standard, and the sequence and timing), was utilized for evaluating bias risk, alongside three domains (selection of participants, the index test, and the reference standard) aimed at identifying concerns regarding applicability [12]. A study was classified as having a high risk of bias if one or more domains were assessed as high risk. Three authors independently appraised the methodological rigor of the included studies using the QUADAS-2 framework. Disagreements were reconciled through collaborative discussion.

### 2.5. Data Analysis

The diagnostic performance measures were analyzed based on the reconstructed 2 × 2 table of true positives (TP), false positives (FP), true negatives (TN), and false negatives (FN) derived from each study. The random-effects models or fixed-effects models were adopted to calculate the diagnostic outcome measures, including the sensitivity, specificity, positive likelihood ratio (PLR), negative likelihood ratio (NLR), diagnostic scores, and odds ratios with their associated 95% confidence interval (CI). To assess the overall efficacy and summarize the diagnostic capability of the MRI and machine learning approach, we generated a summary receiver operating characteristic (SROC) curve and computed the area under the curve (AUC). AUC values were interpreted as follows: ≥ 0.5, 0.75, 0.93, or 0.97 indicating fair, good, very good, or excellent diagnostic accuracy, respectively. Heterogeneity among study results was assessed using Cochran's Q test and the *I*^2^ statistic. Publication bias was investigated through Deeks' funnel plot asymmetry test. For our statistical analyses, we utilized the Midas command within Stata 14.0 (Stata Corp LLP, College Station, TX, USA). The Midas command employs a bivariate mixed-effects logistic regression framework, which is suitable for meta-analyses of diagnostic test accuracy. A *p* value < 0.05 was considered significant.

## 3. Results

### 3.1. Literature Search

**Figure 1:**
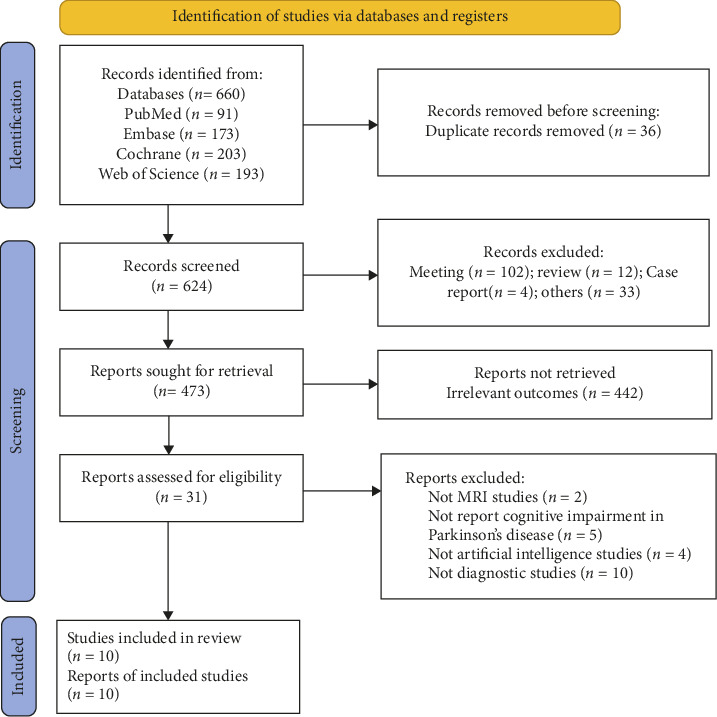
Preferred Reporting Items for Systematic Reviews and Meta-Analyses (PRISMA) study selection flow diagram.

A total of 660 records were identified through a database literature search. After removing duplicates, 36 records were excluded, and 624 records remained for screening. After the screening of the titles and abstracts, 102 meeting abstracts, 12 reviews, four case reports, and 33 other unrelated articles were excluded, and 473 records remained. Furthermore, 442 records were discarded due to irrelevant outcomes, and the full text of the remaining 31 studies was further assessed according to the eligibility criteria. Two studies not employing MRI techniques, five studies not reporting cognitive impairment, four studies not involving artificial intelligence, and 10 studies not focusing on diagnostic approaches were excluded. Finally, 10 studies were ultimately included in this systematic review and meta-analysis (Figure 1).

### 3.2. Characteristics of Included Studies

This present study analyzed 10 studies from 2017 to 2023 on diagnosing PD-MCI using MRI and machine learning, involving 1097 patients from both the training and validation groups. The mean age of these patients ranged from 50 to 70 years [4, 11, 14, 15, 17–22]. These studies employed a variety of machine learning support vector machine (SVM), random forest (RF), XGBoost, decision tree (DT), and neural network (NN). The research was conducted from China, Spain, Korea, Japan, and the UK, highlighting widespread interest in this innovative diagnostic approach (Table 1). The primary outcome of the included studies was PD-MCI, with diagnostic methods for each study detailed in Table 1.

### 3.3. Meta-Analysis Results

**Figure 2:**
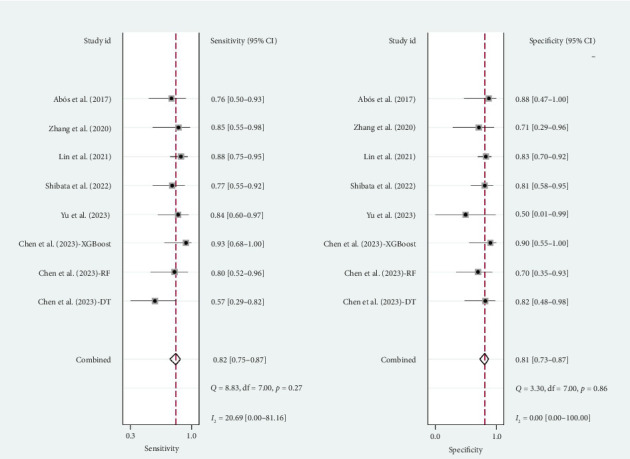
Forest plots of diagnostic sensitivity and specificity of machine learning-assisted MRI in detecting cognitive impairment in Parkinson's disease patients.

**Figure 3:**
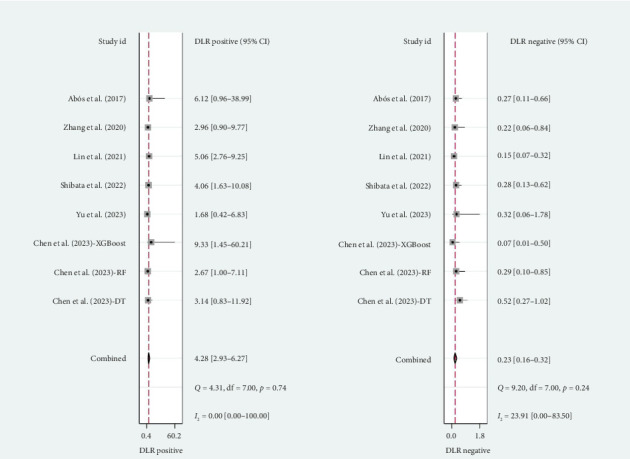
Forest plots of positive and negative diagnostic likelihood ratios for machine learning-assisted MRI in detecting cognitive impairment in Parkinson's disease patients.

**Figure 4:**
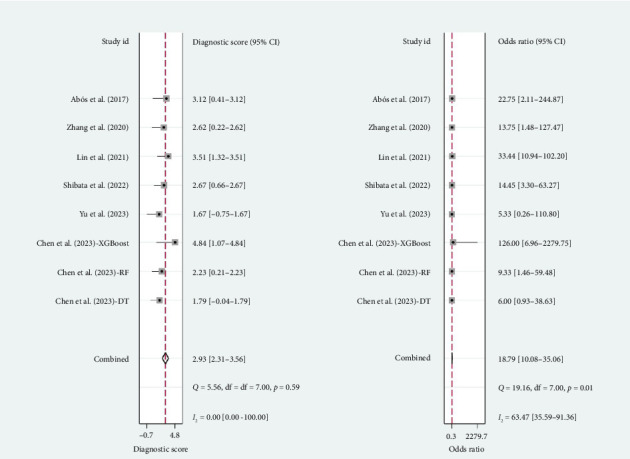
Forest plots of diagnostic score and odds ratio for the efficacy of machine learning-assisted MRI in diagnosing cognitive impairment in Parkinson's disease patients.

**Figure 5:**
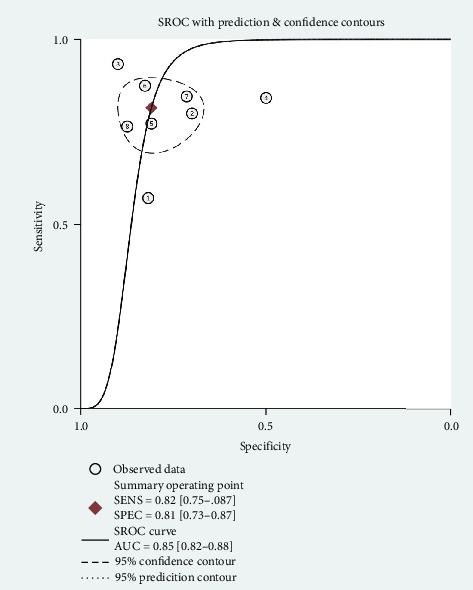
Summary receiver operating characteristic (SROC) curve for machine learning-assisted MRI in diagnosing cognitive impairment in Parkinson's disease.

A total of six studies [4, 11, 14, 17, 20, 22] were included in the meta-analysis, in which the TP, FP, TN, and FN can be obtained. Notably, each of these studies utilized a separate validation group for their models, ensuring that the performance metrics were evaluated on independent data not used during model training. The heterogeneity test for sensitivity (*I*^2^ = 20.69, *p*=0.27) and specificity (*I*^2^ = 0.00, *p*=0.86) suggested minimal heterogeneity among the included studies. The pooled sensitivity and specificity using the fixed-effects models were 0.82 (95% CI: 0.75–0.87) and 0.81 (95% CI: 0.73–0.87), respectively (Figure 2). Figure 2 illustrated the consistency of these metrics across studies, supporting the robustness of machine learning-assisted MRI in diagnosing PD-MCI. The pooled PLR and NLR for machine learning-assisted MRI in detecting PD-MCI, as depicted in Figure 3, were 4.28 (95% CI: 2.93–6.27) and 0.23 (95% CI: 0.16–0.32), respectively. Figure 3 highlights the clinical utility of these ratios, demonstrating that a positive result was more than four times as likely to indicate true cognitive impairment, while a negative result significantly reduced the likelihood of impairment. The analysis shown in Figure 4 presents the forest plots for diagnostic scores and odds ratios, evaluating the efficacy of machine learning-assisted MRI in diagnosing PD-MCI. Figure 4 provides a visual representation of the consistency and variability in diagnostic performance across studies, with each plot contributing to the overall assessment of the diagnostic accuracy of machine learning-assisted MRI. The pooled diagnostic score across the studies was 2.93 (95% CI: 2.31–3.56), using fixed-effects models. For the odds ratios, which measure the odds of correct diagnosis in patients with cognitive impairment versus those without cognitive impairment, the combined value was significantly high at 18.79 (95% CI: 10.08–35.06), also calculated using random-effects models. The AUC for the SROC was 0.85 (95% CI: 0.82–0.88), indicating a high diagnostic accuracy (Figure 5). Figure 5 illustrates the overall performance of machine learning-assisted MRI in distinguishing between patients with and without cognitive impairment, underscoring its potential as a reliable diagnostic tool.

### 3.4. Quality Assessment

As shown in Table 2, seven studies [4, 11, 14, 15, 19, 20, 22] were determined to be at low risk of bias across all domains assessed by the QUADAS-2 tool, while three studies [17, 18, 21] were categorized as high risk of bias. This QUADAS-2 tool was based on the tool's evaluation of four key domains: patient selection, index test, reference standard, and flow and timing.

### 3.5. Publication Bias

**Figure 6:**
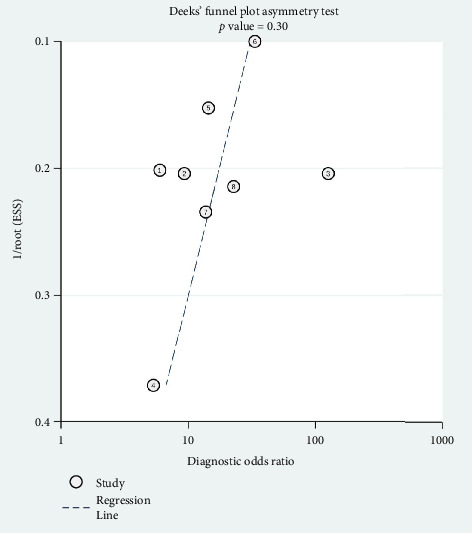
Deeks' funnel plot asymmetry test for publication bias.

The Deeks funnel plot showing the symmetric distribution of the dots with the asymmetry test yielded a *p* value of 0.30, suggesting no significant publication bias within our meta-analysis (Figure 6).

## 4. Discussion

The systematic review and meta-analysis focused on the diagnostic accuracy of machine learning-assisted MRI in detecting PD-MCI. Our comprehensive search strategy across several databases yielded a total of 10 studies that met our inclusion criteria, encompassing a wide array of machine learning models such as SVM, RF, XGBoost, DT, and NN and involving 1097 patients. The meta-analysis of six studies provided robust pooled diagnostic accuracy measures. Specifically, the pooled sensitivity and specificity were found to be 0.82 (95% CI: 0.75–0.87) and 0.81 (95% CI: 0.73–0.87), respectively. These high sensitivity and specificity values indicated that the machine learning-assisted MRI is effective in accurately identifying individuals with PD-MCI, which was crucial for early intervention and management. In a real-world setting, this meant that the approach can reliably detect most cases of PD-MCI while minimizing FPs, thereby facilitating timely therapeutic interventions that may slow a cognitive decline. Furthermore, the clinical significance of these findings lied in their potential to improve patient outcomes by enabling healthcare providers to make informed decisions about treatment strategies tailored to the specific needs of individuals with PD-MCI.

Diagnostic approaches, such as using fecal short-chain fatty acids (SCFAs) as biomarkers for PD-MCI, have shown limited diagnostic accuracy, achieving a sensitivity of 75% and specificity of 66.7% [23]. In contrast, MRI-based diagnostic approaches, particularly when combined with machine learning, offer superior diagnostic accuracy for PD-MCI, providing a more comprehensive and precise assessment of cognitive impairment. One of the key strengths of combining MRI with machine learning is the ability to analyze complex, high-dimensional data to uncover patterns not discernible through conventional methods [17]. The precision of MRI in capturing detailed brain images, combined with the analytical power of machine learning, offers a robust framework for identifying biomarkers of cognitive impairment. Furthermore, this approach facilitates a more objective and quantitative assessment of PD-MCI, reducing the reliance on subjective clinical evaluations [22, 24, 25]. Shibata et al. developed machine learning models trained with QSM values to classify PD patients without dementia into groups with PD-MCI and normal cognition (PD-NC) [4]. The study utilized RF, XGBoost, and RF models for classification and evaluated feature importance within the models. It found that machine learning models could successfully diagnose PD-MCI patients with the accuracy of 79.1%, sensitivity of 77.3%, and specificity of 81.0% [4]. Chen et al. reported machine learning models that classify PD patients into PD-MCI and PD-NC using XGBoost, RF, and DT [11]. The study demonstrated that the XGBoost model achieved the best classification performance, with an accuracy of 91.67% and sensitivity of 92.86% [11]. XGBoost was a DT-based ensemble machine learning algorithm that uses a gradient boosting framework and has proven to be highly effective in handling complex and high-dimensional data, such as those derived from MRI studies [26, 27]. Its ability to efficiently manage missing data, capture nonlinear relationships, and automatically handle feature interactions made it particularly suited for medical diagnostic applications. While these studies showed the promising potential of employing MRI combined with machine learning for diagnosing PD-MCI, it is important to note that many of these studies are limited by relatively small sample sizes. Furthermore, the reported diagnostic accuracies vary across different machine learning models. To the best of our knowledge, this is the first systematic review and meta-analysis focusing on the diagnostic accuracy of machine learning-assisted MRI in detecting PD-MCI, reporting a pooled sensitivity of 82% and specificity of 81%. Furthermore, the pooled PLR was 4.28 (95% CI: 2.93–6.27), indicating that patients with PD-related cognitive impairment are more than four times as likely to have a positive test result with machine learning-assisted MRI compared to those without cognitive impairment. The pooled NLR was 0.23 (95% CI: 0.16–0.32), indicating that if a patient receives a negative result from the machine learning-assisted MRI, the likelihood of them actually having cognitive impairment is reduced to less than a quarter. The integration of this machine learning-assisted MRI approach into clinical settings could significantly enhance diagnostic precision and patient outcomes. By providing a more accurate and objective assessment of PD-MCI, clinicians can make informed decisions about early interventions, potentially slowing cognitive decline and improving quality of life for patients.

There were some limitations in this study. First, the included studies varied in terms of machine learning models used, differences in MRI protocols, and patient demographics. This heterogeneity might impact the pooled results, making it challenging to generalize findings across all PD populations. Specifically, the use of different machine learning algorithms, such as SVM, RF, and XGBoost, could influence the diagnostic accuracy by varying in their ability to handle complex data and capture nonlinear relationships. Second, the included studies had small sample sizes, which might limit the statistical power of the findings. Specifically, small sample sizes could lead to less precise estimates of diagnostic accuracy, making it challenging to draw robust conclusions about the effectiveness of machine learning-assisted MRI in diagnosing PD-MCI. Moreover, the integration of emerging machine learning technique could potentially improve diagnostic accuracy by leveraging their ability to learn complex patterns from larger datasets, thereby offering a promising avenue for future research to enhance the diagnostic capabilities of MRI in PD-MCI. Third, the selection criteria for participants could introduce bias, as studies may prefer participants at particular stages of PD or with specific characteristics, affecting the applicability of the results to the broader PD patient population. Fourth, this meta-analysis included multiple models from a single study, which could introduce bias due to the repeated inclusion of data from the same population. Future research should aim to validate these findings using larger, independent datasets to ensure broader applicability.

## 5. Conclusions

In conclusion, the MRI combined with machine learning for diagnosing PD-MCI achieved high accuracy with the pooled sensitivity of 82% and specificity of 81%. This approach holds significant potential for clinical applications, as it can facilitate early and accurate diagnoses, enabling timely interventions that may improve patient outcomes and quality of life. Future studies should focus on standardizing methodologies and expanding study populations, prioritizing the development of standardized MRI and machine learning protocols to ensure consistency across clinical settings.

## Figures and Tables

**Table 1:** Characteristics of included studies.

Author year	Country	Study design	Gender (% male)	Mean age (years)	Total number (*n*)	Training sample size	Validation sample size	Disease	Gold standard for diagnosis	Disease duration (year)	Validation	Variable selection methods	Model
Abós et al. 2017	Spain	Case-control	58.6	64.7	108	70	25	PD-MCI	UK PD Society Brain Bank Criteria; neuropsychological tests.	10.1	10-fold cross-validation	NR	SVM
Zhang et al. 2020	China	Retrospective cohort	59.1	62	93	73	20	PD-MCI	MDS task force level II; ≥ 1.5 SD below standard neuropsychological scores.	3.1	5-fold cross-validation	NR	SVM
Suo et al. 2021	China	Case-control	46.3	54.3	41	NR	41	PD-MCI	MMSE and MoCA; ≥ 1.5 SDs below normative means.	3.3	NR	NR	SVM
Shin et al. 2021	Korea	Retrospective cohort	47	69	141	117	24	PD-MCI	MoCA; MDS task force Level II criteria.	13	Random sampling	NR	RF/SVM
Lin et al. 2021	China	Retrospective cohort	45.8	62.8	179	131	100	PD-MCI	MoCA; comprehensive neuropsychological assessment according to MDS task force Level II.	9.1	10-fold cross-validation	NR	RF
Shibata et al. 2022	Japan	Retrospective cohort	51.7	72.8	163	120	43	PD-MCI	MDS-UPDRS; MoCA after 12 h without anti-Parkinsonian medications.	8	10-fold cross-validation	NR	RF/XGBoost
Kang et al. 2022	China	Retrospective cohort	50.9	66	149	104	104	PD-MCI	MoCA conducted by two neurologists.	NR	Random sampling	LASSO	LR/SVM
Fiorenzato et al. 2023	UK	Case-control	66.9	63.68	153	118	NR	PD-MCI	MDS-UPDRS-III and HY scale.	9.1	5-fold cross-validation	NR	NN/SVM/RF
Chen et al. 2023	China	Retrospective cohort	53.3	64.4	120	96	24	PD-MCI	MDS task force level I; MoCA < 26 or ≥ 2 neuropsychological tests 2 SDs below control mean.	4.5	10-fold cross-validation	NR	DT/RF/XGBoost
Yu et al. 2023	China	Retrospective cohort	54.3	62.7	90	49	21	PD-MCI	MMSE and MoCA; MDS task force Level I criteria.	4.2	10-fold cross-validation	NR	SVM

*Note:* UK PD Society Brain Bank Criteria: United Kingdom Parkinson's Disease Society Brain Bank Diagnostic Criteria; MDS task force Level 1: movement disorder society task force Level 1; MDS task force Level II: movement disorder society task force Level II.

Abbreviations: HY scale, Hoehn and Yahr scale; MDS-UPDRS, Movement Disorder Society–Unified Parkinson's Disease Rating Scale; MMSE, mini-mental state examination; MoCA, Montreal Cognitive Assessment; SD, standard deviation.

**Table 2:** Risk of bias of the included studies according to the QUADAS-2 tool.

Study	Patients' selection	Index test	Reference standard	Flow and timing	Overall
Abós et al. 2017	High	Low	Low	Low	High
Zhang et al. 2020	Low	Low	Low	Low	Low
Suo et al. 2021	High	Low	Low	Low	High
Shin et al. 2021	Low	Low	Low	Low	Low
Lin et al. 2021	Low	Low	Low	Low	Low
Shibata et al. 2022	Low	Low	Low	Low	Low
Kang et al. 2022	Low	Low	Low	Low	Low
Fiorenzato et al. 2023	High	Low	Low	Low	High
Chen et al. 2023	Low	Low	Low	Low	Low
Yu et al. 2023	Low	Low	Low	Low	Low

## Data Availability

The data that support the findings of this study are available in the Supporting Information of this article.

## Supporting Information

Supporting Table 1: Search strategies in medical databases included.
