# Supplementary material for: Diagnostic Accuracy of Machine Learning-Assisted MRI for Mild Cognitive Impairment in Parkinson's Disease: A Systematic Review and Meta-Analysis
Source: Parkinsons Dis. 2025 May 22;2025:2079341. doi: 10.1155/padi/2079341 (PMC12122149; doi:10.1155/padi/2079341)
Supplement: Supporting Information — Additional supporting information can be found online in the Supporting Information section. [file 2079341.f1.docx]

**Supplementary Table1: Search strategies in medical databases included**

**Search strategy of PubMed**

| NO. | Search Details | Results |
| --- | --- | --- |
| #10 | #9 and #6 | 91 |
| #9 | #7 OR #8 | 216,044 |
| #8 | (((((Artificial Intelligence[MeSH Terms]) OR (Machine Learning[MeSH Terms])) OR (Deep Learning[MeSH Terms])) OR (Supervised Machine Learning[MeSH Terms])) OR (Support Vector Machine[MeSH Terms])) OR (Unsupervised Machine Learning[MeSH Terms]) | 189,620 |
| #7 | ("Magnetic resonance imaging"[MeSH Terms] OR "MRI") AND ("machine learning" OR "Transfer Learning" OR "Deep learning" OR "learning transfer" OR "Ensemble Learning" OR "artificial intelligence" OR "Prediction model" OR "random forest" OR "artificial neural network" OR "ANN" OR "Support vector machine" OR "SVM" OR "Gradient Boosting Machine" OR "GBM" OR "Nomogram" OR "XGboost" OR "Decision tree" OR "Development and validation" OR "risk prediction" OR "risk prediction") | 35,541 |
| #6 | (#5 OR #4) and #3 | 9,252 |
| #5 | "Cognitive Dysfunction" OR "Cognitive Dysfunctions" OR "dysfunctions cognitive" OR "Cognitive Impairments" OR "Cognitive Impairment" OR "impairment cognitive" OR "impairments cognitive" OR "Mild Cognitive Impairment" OR "cognitive impairment mild" OR "cognitive impairments mild" OR "impairment mild cognitive" OR "impairments mild cognitive" OR "Mild Cognitive Impairments" OR "Mild Neurocognitive Disorder" OR "disorder mild neurocognitive" OR "disorders mild neurocognitive" OR "neurocognitive disorder mild" OR "neurocognitive disorders mild" OR "Cognitive Decline" OR "Cognitive Declines" OR "decline cognitive" OR "declines cognitive" OR "Mental Deterioration" OR "deterioration mental" OR "Mental Deteriorations" OR "cognitive defect" OR "cognition disorder" OR "cognition disorders" OR "cognitive defects" OR "cognitive deficit" OR "cognitive disability" OR "cognitive disorder" OR "cognitive disorders" OR "Cognitive Dysfunction" OR "Cognitive Impairment" OR "overinclusion" OR "response interference" | 190,996 |
| #4 | Cognitive Dysfunction[MeSH Terms] | 39,254 |
| #3 | #1 OR #2 | 141,688 |
| #2 | "Parkinson Disease" OR "Parkinson's Disease" OR "Primary Parkinsonism" OR "Parkinsonism, Primary" OR "Paralysis Agitans" OR "idiopathic parkinsonism" OR "paralysis agitans" OR "Parkinson dementia complex" OR "Parkinsons disease" OR "primary parkinsonism" | 141,688 |
| #1 | "parkinson disease"[MeSH Terms] | 84,616 |

**Search strategy of EMBASE**

| No. | Query | Results |
| --- | --- | --- |
| #5 | #3 AND #4 | 173 |
| #4 | 'mri':ti,ab,kw OR 'magnetic resonance imaging':ti,ab,kw | 779756 |
| #3 | #1 AND #2 | 1661 |
| #2 | 'cognitive dysfunction':ti,ab,kw OR 'cognitive dysfunctions':ti,ab,kw OR 'dysfunctions cognitive':ti,ab,kw OR 'cognitive impairments':ti,ab,kw OR 'cognitive impairment':ti,ab,kw OR 'impairment cognitive':ti,ab,kw OR 'impairments cognitive':ti,ab,kw OR 'mild cognitive impairment':ti,ab,kw OR 'cognitive impairment mild':ti,ab,kw OR 'cognitive impairments mild':ti,ab,kw OR 'impairment mild cognitive':ti,ab,kw OR 'impairments mild cognitive':ti,ab,kw OR 'mild cognitive impairments':ti,ab,kw OR 'mild neurocognitive disorder':ti,ab,kw OR 'disorder mild neurocognitive':ti,ab,kw OR 'disorders mild neurocognitive':ti,ab,kw OR 'neurocognitive disorder mild':ti,ab,kw OR 'neurocognitive disorders mild':ti,ab,kw OR 'cognitive decline':ti,ab,kw OR 'cognitive declines':ti,ab,kw OR 'decline cognitive':ti,ab,kw OR 'mental deterioration':ti,ab,kw OR 'deterioration mental':ti,ab,kw OR 'mental deteriorations':ti,ab,kw OR 'cognitive defect':ti,ab,kw | 204447 |
| #1 | 'parkinson disease':ti,ab,kw OR 'parkinsonism, primary':ti,ab,kw OR 'idiopathic parkinsonism':ti,ab,kw OR 'paralysis agitans':ti,ab,kw OR 'parkinson dementia complex':ti,ab,kw OR 'parkinsons disease':ti,ab,kw OR 'primary parkinsonism':ti,ab,kw | 24048 |

**Search strategy of Cochrane Controlled Register of Trials (CENTAL)**

| NO. | Search deatiles | Hits |
| --- | --- | --- |
| #1 | (parkinson disease):ti,ab,kw OR (Parkinson's Disease):ti,ab,kw OR (Primary Parkinsonism):ti,ab,kw OR (Parkinsonism, Primary):ti,ab,kw OR (Paralysis Agitans):ti,ab,kw | 12895 |
| #2 | (idiopathic parkinsonism):ti,ab,kw OR (Parkinson dementia complex):ti,ab,kw OR (Primary Parkinsonism):ti,ab,kw OR (Parkinsons disease):ti,ab,kw OR (primary parkinsonism):ti,ab,kw | 1659 |
| #3 | #1 OR #2 | 12966 |
| #4 | (Cognitive Dysfunction):ti,ab,kw OR (Cognitive Dysfunctions):ti,ab,kw OR (dysfunctions cognitive):ti,ab,kw OR (Cognitive Impairments):ti,ab,kw OR (Cognitive Impairment):ti,ab,kw | 24303 |
| #5 | (impairment cognitive):ti,ab,kw OR (impairments cognitive):ti,ab,kw OR (Mild Cognitive Impairment):ti,ab,kw OR (cognitive impairment mild):ti,ab,kw OR (cognitive impairments mild):ti,ab,kw | 19163 |
| #6 | (impairment mild cognitive):ti,ab,kw OR (impairments mild cognitive):ti,ab,kw OR (Mild Cognitive Impairments):ti,ab,kw OR (Mild Neurocognitive Disorder):ti,ab,kw OR (disorder mild neurocognitive):ti,ab,kw (Word variations have been searched) | 6089 |
| #7 | (cognitive disability):ti,ab,kw OR (cognitive disorder):ti,ab,kw OR (cognitive disorders):ti,ab,kw OR (Cognitive Dysfunction):ti,ab,kw OR (Cognitive Impairment):ti,ab,kw | 52652 |
| #8 | #4 OR #5 OR #6 OR #7 | 53702 |
| #9 | #3 and #8 | 1666 |
| #10 | (Magnetic resonance imaging):ti,ab,kw OR (MRI):ti,ab,kw | 46613 |
| #11 | #9 and #10 | 203 |

Search strategy of web of science

| NO. | Search deatiles | Hits |
| --- | --- | --- |
| #1 | (((((((((TS=(parkinson disease)) OR TS=(Parkinson's Disease)) OR TS=(Primary Parkinsonism)) OR TS=(Parkinsonism, Primary)) OR TS=(Paralysis Agitans)) OR TS=(idiopathic parkinsonism)) OR TS=(paralysis agitans)) OR TS=(Parkinson dementia complex)) OR TS=(Parkinsons disease)) OR TS=(primary parkinsonism) | 196,570 |
| #2 | ((((((((((((((((((((((((((((((((((((TS=(Cognitive Dysfunction)) OR TS=(Cognitive Dysfunctions)) OR TS=(dysfunctions cognitive)) OR TS=(Cognitive Impairments)) OR TS=(Cognitive Impairment)) OR TS=(impairment cognitive)) OR TS=(impairments cognitive)) OR TS=(Mild Cognitive Impairment)) OR TS=(cognitive impairment mild)) OR TS=(cognitive impairments mild)) OR TS=(impairment mild cognitive)) OR TS=(impairments mild cognitive)) OR TS=(Mild Cognitive Impairments)) OR TS=(Mild Neurocognitive Disorder)) OR TS=(disorder mild neurocognitive)) OR TS=(disorders mild neurocognitive)) OR TS=(neurocognitive disorder mild)) OR TS=(neurocognitive disorders mild)) OR TS=(Cognitive Decline)) OR TS=(Cognitive Declines)) OR TS=(decline cognitive)) OR TS=(declines cognitive)) OR TS=(Mental Deterioration)) OR TS=(deterioration mental)) OR TS=(Mental Deteriorations)) OR TS=(cognitive defect)) OR TS=(cognition disorder)) OR TS=(cognition disorders)) OR TS=(cognitive defects)) OR TS=(cognitive deficit)) OR TS=(cognitive disability)) OR TS=(cognitive disorder)) OR TS=(cognitive disorders)) OR TS=(Cognitive Dysfunction)) OR TS=(Cognitive Impairment)) OR TS=(overinclusion)) OR TS=(response interference) | 465,610 |
| #3 | #1 and #2 | 21,910 |
| #4 | (TS=(MRI)) OR TS=(Magnetic resonance imaging) | 632,774 |
| #5 | ((((((((((((((((((ALL=(machine learning)) OR ALL=(Transfer Learning)) OR ALL=(Deep learning)) OR ALL=(learning transfer)) OR ALL=(Ensemble Learning)) OR ALL=(artificial intelligence)) OR ALL=(Prediction model)) OR ALL=(random forest)) OR ALL=(artificial neural network)) OR ALL=(ANN)) OR ALL=(Support vector machine)) OR ALL=(SVM)) OR ALL=(Gradient Boosting Machine)) OR ALL=(GBM)) OR ALL=(Nomogram)) OR ALL=(XGboost)) OR ALL=(Decision tree)) OR ALL=(Development and validation)) OR ALL=(risk prediction) | 4,126,080 |
| #6 | #3 and #4 and #5 | 193 |
